# Infant and child health and healthcare before and after COVID-19 pandemic: will it be the same ever?

**DOI:** 10.1186/s43054-020-00039-7

**Published:** 2020-08-04

**Authors:** Mortada El-Shabrawi, Fetouh Hassanin

**Affiliations:** 1 Faculty of Medicine, Cairo University, Cairo, Egypt; 2 Misr International University, Cairo, Egypt

**Keywords:** COVID-19, Children, Pandemic, Child health, Child healthcare

## Abstract

**Background:**

The novel corona virus disease 2019 (COVID-19) current pandemic is an unpreceded global health crisis. Not only infection of infants, children, and adolescents is a concern for their families and pediatricians, but there are also other serious challenges that should be properly identified and managed as well.

**Main body:**

We have to identify and assess the different factors that have either direct or indirect effects on child health and healthcare due to COVID-19 pandemic and focus on the serious effects. It is easily realized that there are many challenging problems associated with COVID-19 with short-term effects that already appeared and need urgent solutions and long-term effects that are not yet well apparent and have to be searched for and properly addressed.

**Conclusions:**

COVID-19 crisis has lots of impacts on child health and child healthcare, not only from the medical aspect but also from the social, psychological, economic, and educational facets. All these adverse implications have to be identified and dealt with on individual bases approach in the short and long term.

## Background

Since reporting of the first index cases of infection with the novel severe acute respiratory syndrome corona virus 2 (SARS-CoV-2) in Wuhan (Hubei, China) on 12 December 2019, the whole world has changed very rapidly and dramatically. On 30 January 2020, the World Health Organization (WHO) has declared the novel corona virus disease 2019 (COVID-19) as a Global Health Emergency, and shortly thereafter on 11 March 2020, it was declared as a pandemic [1]. COVID-19 pandemic proved rapidly to be a major international medical problem that has many sequences on infants, children, and adolescents. Worldwide, concerted efforts must be exerted in order to identify the huge problems and impacts the pandemic has created that affect child health and child healthcare, and plan prompt solutions for them. All the news, reports, and experiences from the four corners of the world are indicating that infant and child health and healthcare systems before the COVID-19 pandemic have been changed to variable degrees and will probably never be the same after the pandemic in many aspects for extended periods of mankind life.

## Main text

### COVID-19 manifestation in infected children

COVID-19 is uncommon to cause marked clinical symptoms in healthy children as compared with adults. However, asymptomatic children are able to transmit the virus to their adult contacts, and very young infants and children (as well as those with underlying comorbidities) are at increased risk to manifest severe illness [2]. COVID-19 is a droplet infection that spreads rapidly to the unprotected contacts from an infected person. The infectious virus can persist on contaminated surfaces for variable times. The risk of transmission via touching contaminated paper is low, while respiratory and fecal specimens can maintain infectivity for quite a long time at room temperature. SARS-CoV-2 could exist in the air in poorly ventilated buses for at least 30 min. Absorbent materials like cotton are safer than non-absorptive materials for protection from viral infection [3]. As children are less likely to present with serious symptoms, they may have nasal congestion, sore throat, muscular and bony aches, abdominal pain, vomiting, or diarrhea [4]. In children, common circulating corona viruses can cause common cold symptoms such as fever, rhinitis, otitis, pharyngitis, laryngitis, headache, bronchitis, bronchiolitis, wheezing, pneumonia and, in up to 57% of cases, gastrointestinal symptoms (which are more common in children than adults) [5]. Some recent studies have shown that there is limited spread among children and from children to adults [6–8]. The most common manifestations in infected adults include fever, tiredness, and a dry cough [2]. In the majority of infected adults, the symptoms are mild, and more than 85% completely recover. However, the remainder may become seriously ill and some may die. More severe symptoms include difficulty in breathing, pneumonia, acute respiratory distress syndrome, and septic shock leading to multiple organ failure such as heart, liver, and kidney failure [2]. Until now, there is limited evidence that maternal vertical transmission can occur, and newborn infection if occurs is due to perinatal transmission rather than prenatal [9]. It was also found that there is no transmission of the virus through breast milk; therefore, cessation of breast feeding from COVID-19-infected mothers is not recommended, and infected mothers are strictly advised to follow preventive precautions such as handwashing, cleaning the breast before feeding, and using masks during breast feeding [10]. Laboratory findings from children are rather similar to those in adults and include a white blood cell count that is typically normal or reduced with decreased neutrophil count and/or lymphocyte counts. Thrombocytopenia may occur. C-reactive protein (CRP) and procalcitonin levels are often normal. In severe cases, elevated liver enzymes, lactate dehydrogenase levels, as well as an abnormal coagulation and elevated serum ferritin and D-dimers have been reported [5]. Radiologic findings in children are similar to those of adults. Chest radiography mostly shows bilateral patchy airspace consolidations mainly at the periphery of the lungs, peri-bronchial thickening, and ground-glass opacities. Chest computed tomography (CT) scans mostly show airspace consolidations and ground-glass opacities [5].

### Therapeutic and preventive agents

Until now, there is no definitive evidence-based drug therapy for COVID-19 neither in adults nor in the pediatric populations. Current management for COVID-19 is largely symptomatic and supportive care. Supportive measures include sufficient fluid and caloric intake, antipyretics, oxygenation, anticoagulants, and prophylactic antimicrobial therapy to prevent superadded bacterial or fungal infections. The aim is to stabilize the clinical condition and prevent further deterioration as organ failure and secondary infections. It is better for children with mild symptoms to stay at home under medical supervision. If the child condition is deteriorating, then the child should be hospitalized as advised by the treating pediatrician [11].

### Avoiding rumors

Confirmation of the information credibility is essential for healthcare professionals and the public in general. During crisis, rumors and false stories, misleading information, and unreliable data are sadly shared via social media leading to a state of instability and uncertainty among the community members and causing mistrust in the healthcare providers [12]. Pediatricians and all other healthcare team must be cautious about starting therapies based on news or social media reports and should rely on trusted sources of evidence-based information from reliable credited sources of updated information and share those with the families in their care [13].

### Avoiding sham remedies

It is equally important that families be aware that many of what is called sham remedies have been promoted to the public. Many sham treatments have been widely disseminated on social media. These include, for example, drinking warm water, gargling with saline or garlic, drinking lemon juice with honey or black seeds, use of specific homeopathic or alternative medicines, and drinking specific alcoholic drinks. None of these remedies have been proven effective in prevention or treatment, and some have been shown to be harmful, and therefore, should not be recommended [14].

### Healthcare systems failure

Healthcare facilities all over the world became suddenly overwhelmed by unexpectedly treating thousands of COVID-19 patients at the same time. This has created marked congestion and an unpreceded chaos in the healthcare facilities especially in the populated regions. This has its adverse impacts and many people and particularly infants and children were and still (until the time of writing this manuscript) unable to get the proper medical care they actually need. Suggested short-term measures have been proposed to the countries all over the world by the WHO in response to COVID-19 pandemic. A comprehensive guidance to countries on the types of actions and adjustments needed to support the response [15].

### Healthcare delivery and telemedicine

There is an urging challenge of how to provide the required healthcare needed by infants and children in due time and place avoiding the possibility to catch SARS-CoV-2 infection if they go to seek medical advice at hospitals or healthcare facilities. The mandatory precautions including the fundamental physical distancing and infection control requirements will affect the traditional routine medical care beside that many parents are afraid to leave homes or do not want to take their child to a medical care facility with a possibility to be infected from other sick children. Therefore, care givers are encouraged to share their worries and information with their pediatricians via phone calls, e-mails, or other social media applications [16]. Telemedicine has been dramatically exploited in the past few months as a useful tool for long-distance clinical care more than 10-folds what has happened to it during the past decade. Telemedicine can be used for education, counselling parents, and health management, and its role is professionally enlarging in many regions such as the USA and Europe, but awaiting further regulatory approvals in other regions such as in Egypt [17]. Telemedicine may be of limited practical application in some low-income countries where resources are limited due to technical, economic, cultural, or geographical factors, but yet it needs to be tried as an alternative to face-to-face communication to get the required medical advice especially in the straight forward medical problems and concerns. With appropriate attention and caution for some issues such as patient safety, confidentiality, and suspected missed clinical information, telemedicine can be an effective way to help patients during the present COVID-19 pandemic [16, 18].

### Vaccinations

It is estimated that millions of infants and children worldwide have just missed and will continue to miss their required essential vaccinations with a fear that some vaccine preventable diseases (VPD) may come back as measles and poliomyelitis. The WHO has stressed the importance of maintaining the essential health services during COVID-19 pandemic and identified immunization as a core health service that must be offered to the target chirdren [18]. Special planning and extra ordinary efforts are required to be applied quickly for vulnerable pediatric populations at increased risk of morbidity and mortality as refugees and children under custody. However, it was advised that mass vaccination campaigns should be temporarily halted or postponed to follow recommendations on maintaining proper physical distancing and infection control precautions required to combat COVID-19 transmission during such campaigns [19].

### Psycho-social impacts

COVID-19 crisis has forced governments to close nurseries and schools as well as sports’ clubs and gardens. It is not allowed to travel to areas where recreations can be practiced. Children are not allowed to meet their friends and other relatives. They are locked down at their houses having the same repeated daily routine. Similar to adults, children are likely to suffer anxiety, fear, and other psychological manifestations. Children may experience negative feelings and thoughts such as fear of being hospitalized, taking injections up to a fear of their family member loss, or even their own death. This may present as behavioral disturbances, loss of appetite, sleep problems, nightmares, and many other stress-related disorders. The adolescents are also affected but to a lesser extent than children as adolescents seem to express an excellent ability to manage situations of insecurity and have a better adaptation with the changing circumstances [20].

### Child abuse

In the wake of the global lockdown, schools are closed. Children are not only obliged to stay at home for longer hours and become more vulnerable to domestic violence and other sorts of child abuse, but also there is an anticipated decrease in reporting of child maltreatment cases which includes sexual, physical, and emotional abuse. Adding other adverse factors as parental unemployment and economic burdens will be negatively reflected on providing a safe healthy environment for the children to stay in. It is clear now that the measures which have been taken to control the spread of COVID-19 are causing what may be called a “secondary pandemic” of child neglect and abuse [21].

### Refugees and displaced children

The living conditions in refugee camps, crowded reception centers, or detention facilities are unfortunately a very suitable environment for COVID-19 spread. There is lack of proper healthcare services and sanitary precautions beside the suboptimal physical and medical status of the children at such places. Displaced children are among those with the most limited access to prevention services, testing, treatment, and other essential support. In addition, the pandemic and containment measures are likely to have negative consequences for their safety and education, which were pre-carious even before the outbreak of the disease [22].

### Economic aspects

Many families are struggling with their daily lives. Parents and care givers being out of work or even have already lost their jobs during the pandemic do not have enough financial resources to cope with the many changes occurring. On the other hand, the basic needs of infants, children, and adolescents must be fulfilled. With the world economy sagging into recession, it is feared that this hardship will remain and probably increase over the coming months, if not years [23].

#### COVID-19 dilemma for children with a preceding chronic disease

Being more vulnerable to catch infections, children suffering of chronic diseases are at high risk to get COVID-19 infection. Those children are suffering of marked decrease of their protective mechanisms and inner barriers to combat infections. Not only that, but also if they developed COVID-19 infection, there will be a potential increased risk of deterioration of their clinical status. Prevention is the principal key factor for those children. They should not catch COVID-19 at the first place. They must strictly stay at home avoiding any possibility to catch infection. If COVID-19 infection is suspected, they must seek medical advice promptly. Infants < 1 year of age and children with certain serious underlying conditions appear to be at greater risk for severe disease. The most commonly reported underlying conditions in COVID-19 pediatric patients were chronic pulmonary disease, cardiovascular disease, immunosuppression (e.g., related to cancer, chemotherapy, radiation therapy, hematopoietic cell or solid organ transplant, and high doses of glucocorticoids) [24].

### Infants and children who need hospitalization for issues other than COVID-19

The overwhelming current COVD-19 ongoing disaster should not make us forget other serious medical and surgical diseases and emergencies that children may suffer. Pediatricians and pediatric hospitals must be prepared to provide rapid, efficient, and safe medical management accordingly. In its recent Position Statement, the International Pediatric Association (IPA) has strongly recommended that the primary care and hospital resources for children must be maintained during the current COVID-19 pandemic, in order to ensure addressing the child and adolescent health priorities and providing required health management services for children with more severe COVID-19 manifestations [25].

### Education

The mandatory lockdown and inevitable social distancing measures due to the COVID-19 pandemic has forced the governments in many countries to close nurseries, child care centers, schools, training centers, and higher education facilities as universities and institutions. These closures have affected millions of students worldwide not only retarding their educational aspects, but also adversely affecting their emotional status and well-being. Whenever the schools are reopened, the protection of children and educational facilities is particularly important. Precautions are necessary to prevent the potential spread of COVID-19 in school settings; however, care must also be taken to avoid stigmatizing students and staff who may have been exposed to the virus [26].

### Nutritional aspects

Staying at home for long time and closure of sports clubs and lack of physical activities may eventually result in marked weight increase in children and adolescents and development of obesity problem with all its negative consequences. Pediatricians have to alert parents and care givers for this increasing heath problem during lockdowns. On the other hand, in many developing countries, the opposite may occur; the economic adverse effect of COVID-19 may result in marked decrease in the families’ abilities to ensure enough food supplies for their children resulting in their suffering of undernourishment. Nutritious food intake has to be offered to every individual. Proper nutrition and hydration are vital for health these days. Intake of more water and avoiding sugars are essential. Children and adolescents should eat a variety of fresh and unprocessed foods every day to get enough vitamins and minerals [27].

### What is expected after COVID-19 pandemic is over?

The COVID-19 pandemic caused an unpreceded disturbance in the global health systems. Humanity is hopeful that it may come to an end sooner rather than later especially if an effective antiviral treatment(s) and/or vaccine(s) are developed rapidly. Until that moment, prevention of infection and symptomatic and supportive treatment are the best to do. Therefore, revising infant and child health and healthcare plans, and prioritizing the healthcare projects are essential and mandatory issues as the world will never be the same again. Both globalization and urbanization that have been two of the world’s most powerful drivers in the past few decades are anticipated to be reversed by COVID-19 leading to increasing the distances among people and between countries due to border closures and restricted international travel [28].

## Conclusion

So far, the COVID-19 crisis has had a great impact on child health and healthcare all over the world, not only from the medical aspect, but also from the social, psychologic, economic, and educational aspects. All these implications have to be identified and dealt with properly to avoid their short- and long-term consequences on an individual bases approach.

## Data Availability

Not applicable

## Abbreviations

COVID-19: Corona virus disease 2019
WHO: World Health Organization
CRP: C-reactive protein
VPD: Vaccine preventable diseases
IPA: International Pediatric Association
